# Supplementary material for: Controlling the counterintuitive optical repulsive thrust of nano dimers with counter propagating type waves and background medium
Source: PLoS One. 2023 Dec 21;18(12):e0295679. doi: 10.1371/journal.pone.0295679 (PMC10734998; doi:10.1371/journal.pone.0295679)
Supplement: S1 File — (DOCX) [file pone.0295679.s001.docx]

**Supporting information of “Controlling the Counterintuitive Optical Repulsive Thrust of Nano Dimers with Counter Propagating Type Waves and Background Medium”**

SUDIPTA BISWAS^a,b^^, M.R.C. MAHDY^b*^^, SAIKAT CHANDRA DAS^b,c^^,

MD. ARIFUL ISLAM BHUIYAN^b^, MOHAMMAD ABIR TALUKDER^b^

^a^Department of Electrical and Computer Engineering, Baylor University, Waco, Texas, USA.

^b^Department of Electrical & Computer Engineering, North South University, Bashundhara, Dhaka, 1229, Bangladesh.

^c^Abbe School of Photonics, Friedrich Schiller University Jena, Albert-Einstein-Str. 6, 07745 Jena, Germany.

*[mahdy.chowdhury@northsouth.edu](mailto:mahdy.chowdhury@northsouth.edu)

^ These authors have contributed equally.

**S1: Electric Field Profile: Plasmonic-Chiral hybrid dimers illuminated by single plane wave**


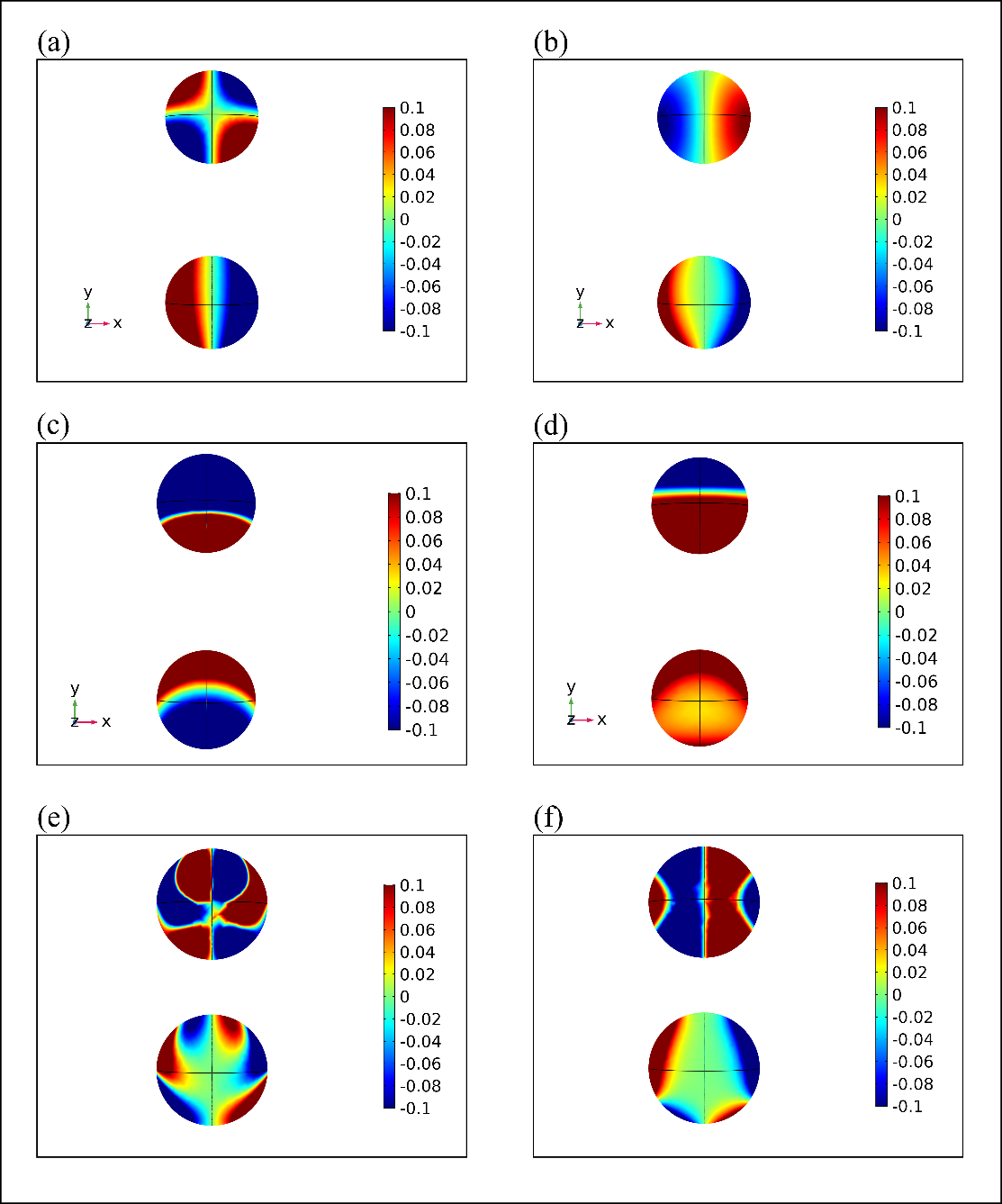


Fig. S1: (a-f) Electric field profiles for configuration of fig. 1(a-c) and interparticle gap distance, d = 200nm (a-b) attractive behavior at wavelength 600nm and 900nm for fig. 1(a) (c-d) repulsive behavior at wavelength 520nm and 790nm for fig. 1(b) (e-f) attractive behavior at wavelength 450nm and 700nm for fig. 1(c).

**S2: Trapping Force of Chiral Nanoparticle**

The Chiral nanoparticle trapping force plays a significant role in the overall reversal of finding force for the proposed set-up. Fig. S2(a-b) shows the F_y_ force of the chiral nanoparticle when the interparticle distance is d=200nm and 700nm, respectively. The polarization of both the lights, in this case, is towards x-direction. From fig. S2(a) we observe that when the overall binding force of the setup reverses at 650nm, the F_y_ force of the chiral particle also becomes negative from positive, i.e., reverses. We also observe a similar trend of the force of the chiral particle for distance d=700nm in fig. S2(b). It is to be noted for fig. S2(a) between wavelength 650-700nm and for fig. S2(b) 850-900nm, the chiral nanoparticle experiences trapping force.


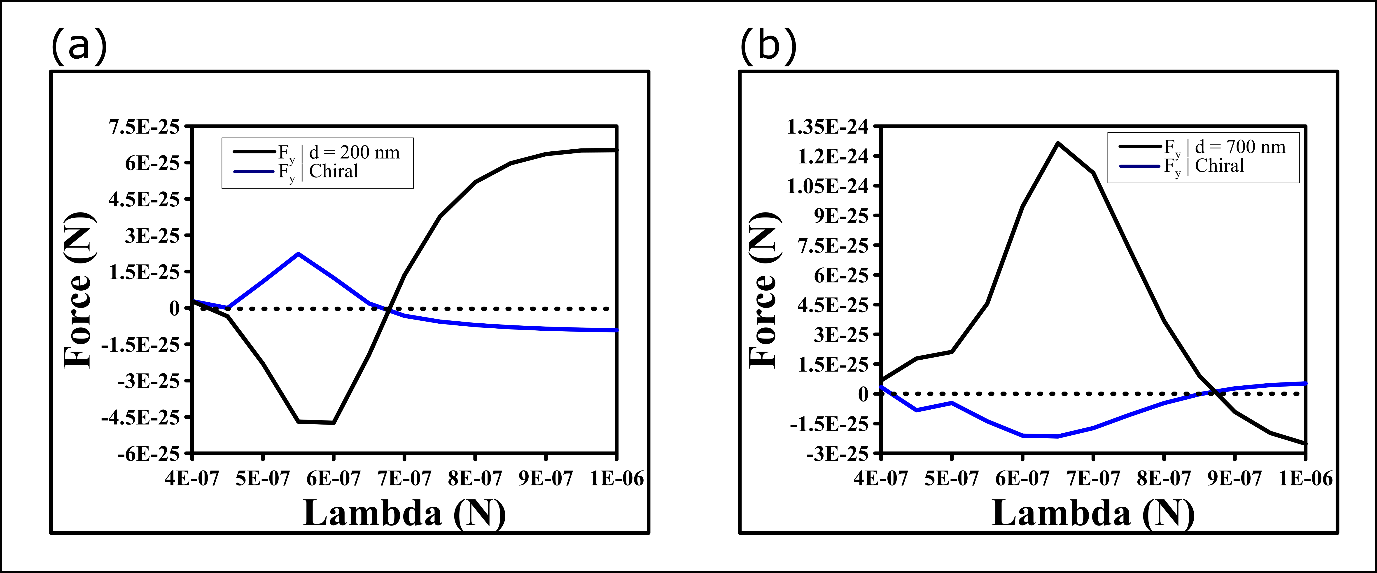


Fig. S2: (a-b) shows the F_y_ force for the chiral nanoparticle and the overall binding force of the configuration for interparticle distance d=200 and 700nm. Both lights, in this case, are polarized towards x-direction.

**S3: Behavior of Chirality in interfering Wave**

For chiral nanoparticles (used in this article), the fundamental relations are given by the following constitutive relations [1,2**]**:

$\left[ \begin{matrix} \mathbf{D} \\ \mathbf{B} \end{matrix} \right]\mathbf{=}\left[ \begin{matrix} \varepsilon_{r}\varepsilon_{0} & i\kappa/c \\ i\kappa/c & \mu_{r}\mu_{0} \end{matrix} \right]\left[ \begin{matrix} \mathbf{E} \\ \mathbf{H} \end{matrix} \right]$ **(S**1)

In order to characterize the local chirality of a monochromatic field, the quantity of chirality density $\mathbf{K}=\mathbf{K}_{\mathbf{e}}+ \mathbf{K}_{\mathbf{m}}= \frac{\varepsilon_{0}}{2} \mathbf{E}\bullet\boldsymbol{\nabla} \times\mathbf{E}+\frac{\mu_{0}}{2}\mathbf{H}\bullet\boldsymbol{\nabla}\times\mathbf{H}$ is introduced [3-5]. $E$ and $H$ are the time-dependent electric and magnetic field of a monochromatic light. Making an analogy to the energy density and energy flow density in Poynting theorem, the chirality flow density is defined as $\boldsymbol{\Phi}=\boldsymbol{\Phi}_{\mathbf{e}}+\boldsymbol{\Phi}_{\mathbf{m}}=\frac{1}{2}\mathbf{E}\times\left( \boldsymbol{\nabla}\times\mathbf{H} \right)-\frac{1}{2}\mathbf{H}\times(\boldsymbol{\nabla}\times\mathbf{E})$ which satisfies the continuity equation $\frac{\delta K}{\delta t}+\nabla\bullet\Phi=0$. $K$ and $\Phi$ are both time independent, suppose in free space, by using Maxwell’s equation we get their time average expressions.

$\bar{\mathbf{K}}=\frac{\omega}{2}\varepsilon_{0}\mu_{0}Im[\mathbf{E}_{\mathbf{0}}\boldsymbol{\bullet}\boldsymbol{H}_{\boldsymbol{0}}^{\boldsymbol{*}}]$ (S2)

$\bar{\boldsymbol{\Phi}}=-\frac{\omega}{4}Im[\varepsilon_{0}\mathbf{E}_{\mathbf{0}}\boldsymbol{\times}\boldsymbol{E}_{\boldsymbol{0}}^{\boldsymbol{*}}+\mu_{0}\boldsymbol{H}_{\boldsymbol{0}}\boldsymbol{\times}\boldsymbol{H}_{\boldsymbol{0}}^{\boldsymbol{*}}]$ (S3)

$E_{0}$ and $H_{0}$ are the standard complex expression of the field. The chirality density K and chirality flow density $\Phi$ are actually the energy and momentum operators of the field multiplied by the helicity h [5]. The chirality flow density is also direct related to the SAM density [5].

In this case, the electric field consists of counter-propagating plane waves with orthogonal polarizations. We write the total field as

$\tilde{\boldsymbol{E}}=E_{inc}\exp\left( \mathrm{iky} \right)\hat{\boldsymbol{x}}+E_{inc}exp(-iky)\hat{\boldsymbol{x}}$ (S4)

The ellipticities of the electric and magnetic field are $\sigma_{e}=-\sin\left( 2ky \right)$ and $\sigma_{m}=\sin\left( 2ky \right)$ respectively. $\sigma_{e}$ and $\sigma_{m}$ are always opposite number indicating the electric field and the magnetic field rotate in opposite sense at any position.

Substituting the field into (1), we find the chirality density $\bar{K}$ is zero everywhere. The chirality flow is

$\bar{\boldsymbol{\Phi}}=\bar{\boldsymbol{\Phi}_{\mathbf{e}}}+\bar{\boldsymbol{\Phi}_{\mathbf{m}}}=-\frac{1}{2}\omega\varepsilon_{0}E_{inc}^{2}\sin\left( 2ky \right)+\frac{1}{2}\omega\mu_{0}H_{inc}^{2}sin(2ky)$ (S5)

Although the electric $\bar{\Phi_{e}}$ and magnetic components $\bar{\Phi_{m}}$ of the chirality flow are non-zero, the total chirality flow ($\bar{\Phi}$) is zero since both the component contribute equality to the total flow and always flow in opposite directions. This equal contribution fulfills the principle of dual ‘electric-magnetic’ symmetry of Maxwell equation in free space. Being different from the

field, matter is strongly dual-asymmetric. As a result, in the light-matter interaction, the electric and magnetic parts of the field may contribute very differently in term of momentum transfer. This unequal contribution leads to the trapping force of chiral particle.

**S4: Plasmonic Chiral Hybrid Heterodimers put in the air (homogeneous) medium in presence of counter propagating waves: Face to Face Repulsive Force for Particles in Dipolar Range.**

In the previous case, we observed the reversal of OBF for particles lying in the Rayleigh region with a second source of a linearly polarized plane wave. Following the previous case, we decided to explore the idea of reversal of the near- and far-field OBF of a plasmonic chiral heterodimer in the dipolar region. In fig. 2(c), we could not achieve reversal of OBF for particles lying in the Dipolar region by applying a single light source. Similar to the previous case, we were able to acquire reversal of longitudinal OBF with the addition of a second linearly polarized plane wave propagating in the opposite direction in this case as well. The propagation and polarization of light are similar to the previous case. The wavelength (λ) of the lights ranges from 400 nm to 1000 nm.

Fig. S3(a) shows our new configuration after adding a second source light source and fig. S3(b) shows the achieved OBF using this new arrangement for interparticle distance 200nm and 700nm. From fig. S3(c), we observe that for interparticle distance d=200nm, the optical force at lower wavelength is attractive. The maximum attractive force is observed at λ=550nm. However, at λ=650nm the force reversal and becomes repulsive. The repulsive force reaches its peak at λ=950nm. Similar reversal of longitudinal OBF is observed for other configurations as well.


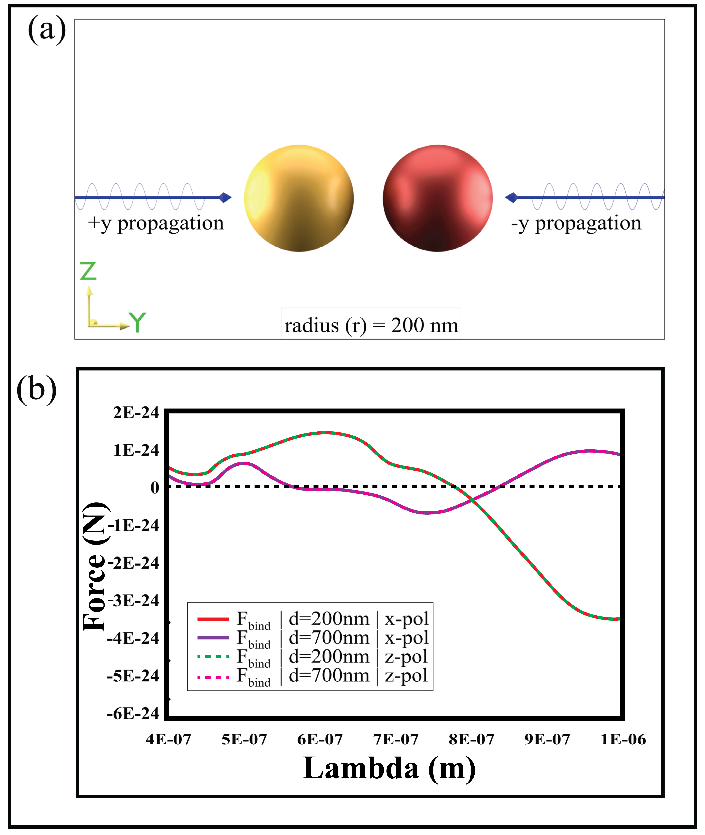


Fig. S3: (a) “x” or “z” polarized plane waves are propagating towards both “+y” and “-y” directions hitting the plasmonic(left) and chiral(right) nanoparticles from both sides. The particles lie in the Dipolar region, and their radius is r=200nm. (b) The OBFs (along the “y” - axis) are shown between the two-hybrid dimers for fig. 7(a). The interparticle gap distance between the dimers is 200 nm and 700 nm by varying wavelengths. [Left particle is plasmonic and right particle is chiral].

In this case, as well, the particles depict counter-intuitive behavior. The electric field profiles for this case are shown in supplement section S4. From fig. S4(a-h) we get the electric field profiles for fig. S3(a) for interparticle distance 200nm and 700nm. Here, fig. S4(a-b) shows the electric field profiles for interparticle distance, d=200, where the polarization of both the lights is towards x-direction. From fig. S4(a), the F-F charge of the plasmonic and chiral nanoparticles depict attractive behavior when the optical force is attractive [cf. Force results in fig. S3(b)] and shows repulsive behavior when the OBF is repulsive. Consequently, fig. S4(c-d) shows the electric field profile for far-field areas of the above arrangement, while fig. S4(e-h) shows the electric field profiles when the lights are polarized in the z-direction.

We already know from earlier that the presence of two sources of plane waves increases the strength of the electric field manifold. Due to the forming of surface plasmons on the plasmonic, as EM wave propagates through the plasmonic particle creates an effective plasmonic force [6, 7]. This plasmonic force combined with the strong charge flow effect induces stronger coupling and resonance on the nanoparticles, causing the overall binding force to reverse; consequently, it also enables us to achieve a ‘direct’ reversal of longitudinal OBF for particles lying in the Dipolar region.


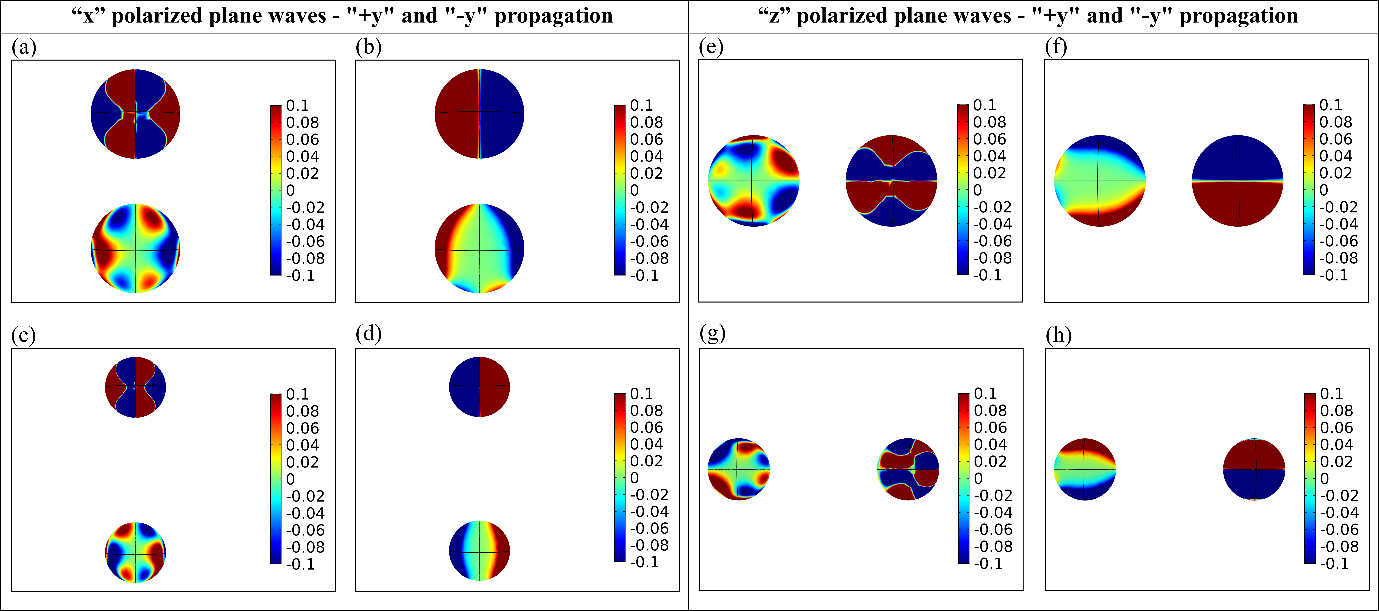


Fig. S4: (a-d) electric field profiles for configuration of fig. 7(a) when “x” polarized plane waves are propagating in the “+y” and “-y” direction. (a) interparticle distance, d = 200 nm at wavelength 450nm (b) interparticle distance, d = 200 nm at wavelength 850nm (c) interparticle distance, d = 700 nm at wavelength 450nm (d) interparticle distance, d = 700 nm at wavelength 900nm. (e-h) electric field profiles for configuration of fig. 7(a) when “z” polarized plane waves are propagating in “+y” and “-y” direction. (e) interparticle distance, d = 200 nm at wavelength 450nm (f) interparticle distance, d = 200 nm at wavelength 850nm (g) interparticle distance, d = 700 nm at wavelength 450nm (h) interparticle distance, d = 700 nm at wavelength 900nm. [The size of the particles is r=200nm]

**S5: Numerical Model of the proposed configuration from COMSOL Multiphysics 5.3A.**


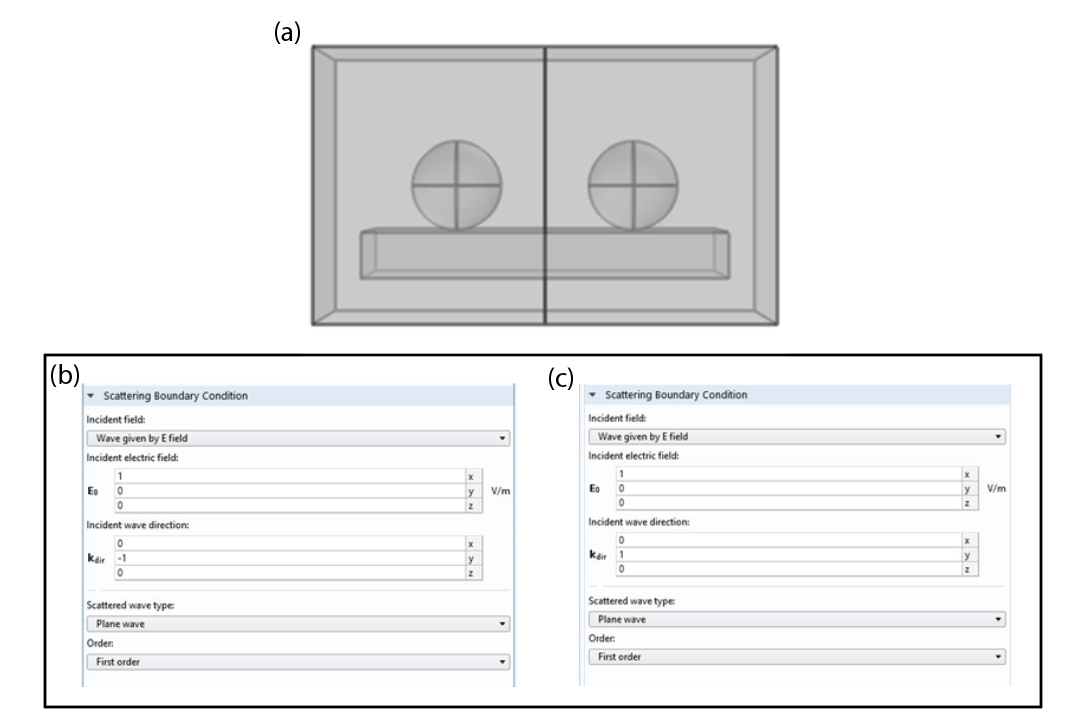


Fig S5: (a) Shows the model from the COMSOL Multiphysics 5.3A. (b) ‘x’ polarized plane wave propagating in “-y” direction (c) ‘x’ polarized plane wave propagating in “+y” direction.

**References:**

1. Deniz, A. A., Mukhopadhyay, S., & Lemke, E. A. (2008). Single-molecule biophysics: at the interface of biology, physics and chemistry. Journal of the Royal Society Interface, 5(18), 15-45.
2. Rahim, Masudur, Saikat Chandra Das, MD Mohaimanul Masud Sunny, and M. R. C. Mahdy. "A generic metasurface for the optical pulling of dielectric or plasmonic or chiral Mie objects." Optics Communications (2021): 127679.
3. Y. Q. Tang, and A. E. Cohen, "Enhanced Enantioselectivity in Excitation of Chiral Molecules by Superchiral Light," Science **332**, 333-336 (2011).
4. K. Y. Bliokh, and F. Nori, "Characterizing optical chirality," Phys Rev A **83** (2011).
5. S. M. Barnett, "Rotation of electromagnetic fields and the nature of optical angular momentum," J Mod Optic 57, 1339-1343 (2010).
6. Mitri, F. G. (2018). Pushing, pulling and electromagnetic radiation force cloaking by a pair of conducting cylindrical particles. Journal of Quantitative Spectroscopy & Radiative Transfer, 206, 142–150.
7. Bohren, C. F., & Huffman, D. R. (2008). Absorption and scattering of light by small particles. John Wiley & Sons.
