# Supplementary material for: Controlling the counterintuitive optical repulsive thrust of nano dimers with counter propagating type waves and background medium
Source: PLoS One. 2023 Dec 21;18(12):e0295679. doi: 10.1371/journal.pone.0295679 (PMC10734998; doi:10.1371/journal.pone.0295679)
Supplement: Attachment — Submitted filename: PONE-D23-24071_review.pdf [file pone.0295679.s002.pdf]

This paper provides a method to control the repulsion and attraction behavior of nanoparticles using a "modified" longitudinal optical binding force. It demonstrates that the broadband, especially in higher wavelength regions, repulsive binding force can be achieved using counterpropagating waves in heterogeneous background, due to the linear increase of momentum of the emitted photons, while it is not achievable in homogeneous background. The theoretical and numerical analysis show corresponding results which support the authors claim. The paper is recommended for publication upon satisfactorily answering the following questions.

1. What is the relation between the optical binding force and the electric polarity and magnetic rotation? At the moment, the repulsive behavior seem to be related to the similarity between the induced electric polarity and magnetic rotation in chiral and plasmonic particles. Is it possible to generate optical binding forces using the set-up in Figs. 1(a) and (b) using plasmonic-chiral dimers? It is important to show that there is actually conventional optical binding force here and then controlling the repulsive thrust can be discussed. The current observation seems to suggest the repulsive behavior comes from particle-particle interaction instead of light-particle interaction.
2. Although it has mentioned several times in the manuscript, it is not clear to the reviewer why in homogeneous case wave nature of light plays a vital role, but in heterogeneous case the particle nature plays a vital role. There lacks discussion which points out explicitly the feature or behavior that relates to wave and particle nature, respectively, based on observations.
3. Basically, the authors have not explained why the car-person interaction is analogy to the light-particle interaction in Fig. 1. When persons move away from each other, they must push the car back as shown in the figures, but when particles move away, the waves cannot be pushed back. I don't see this as an appropriate analogy.
4. It is highly suggested that the authors summarize and itemize the "modification" in the so called "modified" optical binding force.

Other minor points:

1. Fig. 4(b) misses the other two curves although they are listed in the legend. It is suggested to use different line types or markers to plot the curves such that the overlapped data can still be seen from the figure.
2. It is suggested to mark the plasmonic and chiral particle in Fig. 5.
3. It is suggested to substitute all the line plots in Fig. 8 with higher resolution figures with readable values on the axes.
