# Supplementary material for: Controlling the counterintuitive optical repulsive thrust of nano dimers with counter propagating type waves and background medium
Source: PLoS One. 2023 Dec 21;18(12):e0295679. doi: 10.1371/journal.pone.0295679 (PMC10734998; doi:10.1371/journal.pone.0295679)
Supplement: Attachment — Submitted filename: Response Letter_revised.docx [file pone.0295679.s003.docx]

Response Letter

Dear Editor,

We are submitting the revised version of the manuscript ["Controlling the Counterintuitive Optical Repulsive Thrust of Nano Dimers with Counter Propagating Type Waves and Background Medium" (ID: PONE-D-23-24071)] along with the response letter. We have carefully addressed both the reviewers’ comments and made the consequent revisions and improvements of the manuscript. All changes to our main manuscript article have been marked in red color. We believe that: you will really consider the merit of the proposed results and the current version of the overall work.

We’d like to manifest our sincere thanks to the reviewers for their constructive and informative comments to improve our manuscript, all of which are valuable for the improvement of our work. In this response letter, we list all the comments and our replies one by one.

Sincerely yours,

Dr. Mahdy Rahman Chowdhury.

Associate Professor, North South University.

Ph.D., Optical force and manipulation, National University of Singapore.

- On behalf of all the authors.

Reviewer #1

This manuscript introduces a new discovery in the realm of light-matter interaction: the manipulation of the counter-intuitive repulsive force within the proximity and distant regions of plasmonic-chiral heterodimers. This is achieved by subjecting them to linearly polarized counter-propagating plane waves. To influence and manage this repulsive force exerted on the dimers, the authors have explored two different contexts, homogeneous and heterogeneous backgrounds in our configurations.

While I find the content intriguing, I am unable to endorse its publication in its current state. The following points outline my concerns and questions:

1. While the concept and content of this manuscript are innovative, the manner in which it is presented makes it challenging to grasp the overarching structure. The document leans heavily towards a descriptive style, yet the logical flow poses difficulties for readers to follow. The authors may consider revising the article's structure to achieve a more organized and coherent presentation.

Ans: Dear reviewer, thank you very much for your valuable feedback. We have addressed the issues raised and have revised the article's structure to ensure a more organized and coherent presentation. Redundancies have been minimized, and the overall language of the paper has been refined to enhance readability. The newly edited part has been colored yellow for our manuscript.

2. Following 1, it is advisable for the authors to emphasize the underlying physical mechanism or rationale behind this altered longitudinal OBF. Allocating a distinct section to elucidate this aspect could assist readers in concentrating on and comprehending it.

Ans: Dear reviewer, thank you for your suggestion. We have added a completely new section describing underlying physical mechanism or rationale behind this altered longitudinal OBF. The section is titled “***3.1.1 Underlying Physical Mechanism Behind the Altered Longitudinal OBF***” in our main manuscript.

3. The authors should enhance the substantiation of their claim that "By increasing the refractive index of the lower medium, it is indeed possible to further enhance the repulsive thrust for the setups shown in Fig. 8(a) and (h)." as pointed out in line 621.

Ans: Dear reviewer, we apologize for any confusion that may have arisen due to the structure of the article, making it seem as though our claim on line 538 (previously 621), stating “By increasing the refractive index of the lower medium, it is indeed possible to further enhance the repulsive thrust for the setups shown in Fig. 8(a) and (h)," was not sufficiently explained. In reality, we tried to enhance the optical repulsive thrust that we obtained for our full air setup, as depicted in Fig. 4. We accomplished this by changing the lower medium from air to water, thereby increasing the refractive index from n = 1 to n = 1.33. As a result, we got repulsive force on order of magnitude higher [cf. Fig. 8]. To clarify this phenomenon, we have revised the paragraph in our main manuscript as follows:

“Finally, considering the far-field case where the interparticle distance is 700 nm, as depicted in Fig. 8(h)-(j), we observe a similar phenomenon. The broad-band repulsive thrust between the dimers is a result of the individual pulling forces acting on the Rayleigh-sized chiral and plasmonic particles embedded in a heterogeneous background. Our analysis indicates that the physical reasoning for this occurrence aligns closely with the near-field case. The multipolar behavior of the Rayleigh-sized plasmonic particle and the dipolar behavior of the Rayleigh-sized chiral particle are further detailed in Fig. 9(d) - 9(f). This observation substantiates that increasing the refractive index of the lower medium [water (n = 1.33) replaces air (n = 1) as a lower medium] can effectively intensify the repulsive thrust for the configurations illustrated in Fig. 8(a) and (h)”.

4. Despite the authors' inclusion of a 15-degree incident angle in both Fig. 2 and Fig. 8, there are discrepancies apparent in the schematic where certain angles do not conform to 15-degree. It is recommended that the authors address and rectify this inconsistency."
Ans: Dear reviewer, we are grateful for your observant eye in identifying the discrepancies in the schematic representation of the incident angles in Fig. 2 and Fig. 8. We have rectified this inconsistency, ensuring that the angles accurately conform to 15 degrees as intended. The revised figures also included herewith.

Figure 2:

Figure 8:

5. What is the reason behind having four legends in Fig. 4(b) when only two curves are actually shown?

Ans: Dear reviewer, thank you once more for your meticulous attention to the details within our paper. The OBF for both x-pol and z-pol in Fig. 4(b) are similar (which is expected), resulting in their overlap. To address this and enhance clarity, we have modified the figure to provide a more accurate representation of the scenario.

6. The x-axis unit in Fig. 4(b) is labeled incorrectly.
Ans: Dear reviewer, we appreciate your attention to detail and would like to inform you that we have amended the label of Fig. 4(b) accordingly. The initial notation, Lambda (N), has been corrected to Lambda (m). The figure is also attached below.

7. There are some symbols typo such as “k” on line 237 should be “\kappa” and $\bar{P}$ in line 416 should be $\bar{p}$.
Ans: Dear reviewer we express our gratitude for your attentiveness to the details within our manuscript. We have taken immediate action to correct these issues. The symbol “k” on line 237 has been accurately amended to “\kappa”, and likewise, the symbol $\bar{P}$ in line 416 has been corrected to $\bar{p}$.

8. There are grammatical errors in many sentences, such as "In differentiate, it shows up that the wave nature of light is playing a crucial part for the light-matter interaction of our proposed setup."

Ans: Dear reviewer, thank you very much for your insightful feedback. We have proofread the entire manuscript once again and rectified numerous grammatical inaccuracies, including the specific one you have highlighted. Your attention to detail is immensely appreciated, and we hope that these revisions will contribute to a clearer and more coherent presentation of our work.

9. Enhancement of the resolution quality in Fig. 8 is recommended, as the current presentation lacks clarity in showcasing finer details.
Ans: Dear reviewer, thank you for pointing out the clarity issue in Fig. 8. We have acted upon your recommendation and enhanced the resolution to better showcase the finer details.

10. The authors should consider revising their use of abbreviations to avoid repetition. For instance, on page 2, the authors already introduce the abbreviation "OBF" for "optical binding force" on line 97. However, on line 226, instead of utilizing the established abbreviation "OBF," they continue to abbreviate "optical binding force" again.

Ans: Dear reviewer, thank you for bringing this to our attention. We have reviewed the document and rectified the inconsistent use of abbreviations, ensuring that once an abbreviation is introduced, it is used consistently throughout the paper. We appreciate your diligence in helping to improve the clarity of our work.

Reviewer #2

This paper provides a method to control the repulsion and attraction behavior of nanoparticles using a “modified” longitudinal optical binding force. It demonstrates that the broadband, especially in higher wavelength regions, repulsive binding force can be achieved using counterpropagating waves in heterogeneous background, due to the linear increase of momentum of the emitted photons, while it is not achievable in homogeneous background. The theoretical and numerical analysis show corresponding results which support the authors claim. The paper is recommended for publication upon satisfactorily answering the following
questions.

1. What is the relation between the optical binding force and the electric polarity and
magnetic rotation? At the moment, the repulsive behavior seem to be related to the
similarity between the induced electric polarity and magnetic rotation in chiral and
plasmonic particles. Is it possible to generate optical binding forces using the set-up in
Figs. 1(a) and (b) using plasmonic-chiral dimers? It is important to show that there is
actually conventional optical binding force here and then controlling the repulsive thrust
can be discussed. The current observation seems to suggest the repulsive behavior comes
from particle-particle interaction instead of light-particle interaction.

Ans: Dear reviewer, thank you so much for the insight. In our manuscript, we have explored the control of optical binding force (OBF), a phenomenon commonly influenced by the presence of similar or opposite charges on nanoparticles. The fundamental principle here is that like charges attract each other, while opposite charges repel, aligning seamlessly with the concept of OBF. This aspect has been discussed and integrated into our manuscript.

“At a lower wavelength with interparticle distance d=200nm, where the OBF is repulsive, both positive and negative charges accumulate on the plasmonic and chiral nanoparticles, resulting in an overall repulsive force between them [Fig. 5(a)]. Contrastingly, Fig. 5(b) illustrates that at higher wavelengths, where OBF is attractive, the induced electric field exhibits attractive behavior due to the accumulation of opposite charges on the nanoparticles. The electric field profiles for far-field regions of this configuration are depicted in Fig. 5(c-d), and Fig. 5(e-h) displays the profiles when the lights are polarized towards the +z direction for interparticle distances of 200nm and 700nm, respectively.”

Furthermore, another significant factor influencing OBF is the current density profile of plasmonic and chiral particles. Our observations have revealed interesting patterns in the rotational current density associated with these particles concerning the direction of OBF. Specifically, when OBF is attractive, we observe a rotational current density on the plasmonic particle, indicating its stronger influence on the overall binding force. However, this rotational current density diminishes when the binding force reverses. Following the reversal, we notice a rotational current density on the chiral particle, suggesting its heightened impact on the overall OBF. These findings are also mentioned in our manuscript.

“We have identified an additional phenomenon contributing to the reversal of longitudinal OBF. Fig. 6(a-d) illustrates the current density profile for plasmonic and chiral particles, correlating with the configuration in fig. 4(a), where both lights are polarized towards the x-direction. The arrows in fig. 6(a-d) represent the direction of induced electric current flow. Examining fig. 6(a-b), we notice variations in current density for both plasmonic and chiral particles at different wavelengths, with an interparticle distance, d=200 nm. When the OBF is repulsive, a rotating current density pattern is noticeable on the chiral particle, indicative of an induced magnetic type resonance [cf. fig. 6(b)]. Such a phenomenon is absent in the plasmonic particle at these wavelengths. Conversely, in regions where the OBF is attractive, the chiral particle's rotating pattern vanishes, and a rotating pattern emerges on the plasmonic particle, indicating plasmonic type resonance [cf. fig. 6(a)]. Similarly, with an interparticle distance, d=700 nm, a comparable rotating pattern is observed on the particles at various wavelengths, as depicted in fig. 6(c-d).”

It's important to note that we did not observe any significant OBF in our single polarized light cases, as illustrated in Figure 3(d-e). It is only when we introduced two counter-propagating lights that we observed the conventional longitudinal optical binding force.

2. Although it has mentioned several times in the manuscript, it is not clear to the reviewer
why in homogeneous case wave nature of light plays a vital role, but in heterogeneous
case the particle nature plays a vital role. There lacks discussion which points out explicitly
the feature or behavior that relates to wave and particle nature, respectively, based on
observations.

Ans: **Wave Nature:**

From the revised manuscript:

“Certainly, the presence of the other particle in the dimer set is impacting the dynamics of another particle. The generic approach to explain such dynamics based on well-known dipolar force model [35] for the ‘simple’ dipoles:

In this model, the incident field induces a dipole in both the left-side and right-side particles, with the right-side dipole oscillating with a phase shift kd (where d represents the interparticle distance) relative to the left-side one. The right-sided dipole then radiates backward toward the left sided dipole. This backward-scattered light reaches the oscillating left sided dipole with the total phase shift of 2kd. The force on the left sided particle arises from the interaction of the left sided dipole with the interference effect of the backward-scattered field and incident field. On the other hand, the force on the right sided particle pushes the right sided dipole along the wave propagation direction. In this way, the interaction between both ‘simple’ dipoles is usually repulsive in the very closely placed near field region.”

- This clearly supports the wave nature of light.

**Particle Nature:**

From the revised manuscript:

“For our case, the plasmonic particle is experiencing pulling force (towards its nearest unperturbed light source) in the dimer set-up of Fig. 8(a) for the combined impacts of three catalysts together: (i) the presence of high non-paraxial angled beams (that creates multipolar response), (ii) transfer of Minkowski momentum (of emitted photon) to the plasmonic particle and (iii) repulsive force in the near field in presence of the chiral particle due to mutual scattering. If any of the aforementioned catalysts is removed, mutual repulsion at higher wavelength region or individual pulling type force vanishes. In summary, the dimer set, illustrated in Fig. 8(b) and (c), experiences a repulsive binding force even in higher wavelength regions, as depicted in Fig. 8(a), due to the pulling force exerted on each particle. Here, the pulling force refers to the force directing a particle towards the closest unperturbed light source.”

- The Minkowski momentum of photon suggests the linear increase of momentum of light at the interface of the half-immersed object and the background [A]. This increase of the linear momentum of light strongly suggests the existence of photon or the particle nature of light. An experimental observation in favor of such linear increase of momentum of photon for the Mie particle (but not Rayleigh or Dipolar particle) can be found in [B] and its initial analysis can be found in [A, C].

[A] Mahdy, M. R. C., Hamim Mahmud Rivy, Ziaur Rahman Jony, Nabila Binte Alam, Nabila Masud, Golam Dastegir Al Quaderi, Ibraheem Muhammad Moosa, Chowdhury Mofizur Rahman, and M. Sohel Rahman. "Dielectric or plasmonic Mie object at air–liquid interface: The transferred and the traveling momenta of photon." *Chinese Physics B* 29, no. 1 (2020): 014211.

[B] Kajorndejnukul, Veerachart, Weiqiang Ding, Sergey Sukhov, Cheng-Wei Qiu, and Aristide Dogariu. "Linear momentum increase and negative optical forces at dielectric interface." Nature Photonics 7, no. 10 (2013): 787-790.

[C] Qiu, Cheng-Wei, Weiqiang Ding, M. R. C. Mahdy, Dongliang Gao, Tianhang Zhang, Fook Chiong Cheong, Aristide Dogariu, Zheng Wang, and Chwee Teck Lim. "Photon momentum transfer in inhomogeneous dielectric mixtures and induced tractor beams." Light: Science & Applications 4, no. 4 (2015): e278-e278.

3. Basically, the authors have not explained why the car-person interaction is analogy to the
light-particle interaction in Fig. 1. When persons move away from each other, they must
push the car back as shown in the figures, but when particles move away, the waves cannot
be pushed back. I don't see this as an appropriate analogy.

Ans:	Dear reviewer, Thank you so much for your suggestion. We have revised our article carefully and therefore we removed the car-person analogy form our manuscript.

4. It is highly suggested that the authors summarize and itemize the “modification” in the
so called “modified” optical binding force.

Ans: Thank you for your insightful suggestion. We have taken it into consideration and further clarified the distinctions that make the Optical Binding Force (OBF) observed in our study 'modified.' The term 'modified' has been used to denote the variations from the conventional OBF, which commonly occurs with simpler entities under different conditions. This distinction is made in contrast to the conventional longitudinal OBF, typically observed with a single light source or between two simplistic dipolar entities like dielectric dipole-sized objects, in the presence of counter-propagating waves.

We hope this clarification further elucidates the unique nature of the ‘modified’ OBF observed in our study and distinguishes it from the conventional OBF.

“The term “modified” is used due to the presence of two oppositely propagating waves, contrasting with a single propagating wave scenario, with additional reasons detailed in section 4.”

Other minor points:
1. Fig. 4(b) misses the other two curves although they are listed in the legend. It is suggested
to use different line types or markers to plot the curves such that the overlapped data can
still be seen from the figure.

Ans: Dear reviewer, thank you once more for your meticulous attention to the details within our paper. The OBF for both x-pol and z-pol in Fig. 4(b) are similar (which is expected), resulting in their overlap. To address this and enhance clarity, we have modified the figure to provide a more accurate representation of the scenario.

Fig. 4: (a) “x” or “z” polarized plane waves are propagating towards both “+y” and “-y” directions hitting the plasmonic(left) and chiral(right) nanoparticles from both sides. The particles lie in the Rayleigh region, and their radius is r=100nm. (b) The OBFs (along the “y” - axis) are shown between the two-hybrid dimers for fig. 4(a). The gap distance between the dimers is 200 nm and 700 nm by varying wavelengths.

2. It is suggested to mark the plasmonic and chiral particle in Fig. 5.

Ans: Thank you for your constructive suggestion regarding Fig. 5. We agree that marking the plasmonic and chiral particles would enhance the figure's clarity and aid in better understanding. We have accordingly revised Fig. 5 to include clear markings that distinguish between the plasmonic and chiral particles.

Fig. 5: (a-d) electric field profiles for configuration of fig. 4(a) when “x” polarized plane waves are propagating in the “+y” and “-y” direction. (a) interparticle distance, d = 200 nm at wavelength 450nm (b) interparticle distance, d = 200 nm at wavelength 850nm (c) interparticle distance, d = 700 nm at wavelength 450nm (d) interparticle distance, d = 700 nm at wavelength 900nm. (e-h) electric field profiles for configuration of fig. 4(a) when “z” polarized plane waves are propagating in “+y” and “-y” direction. (e) interparticle distance, d = 200 nm at wavelength 450nm (f) interparticle distance, d = 200 nm at wavelength 850nm (g) interparticle distance, d = 700 nm at wavelength 450nm (h) interparticle distance, d = 700 nm at wavelength 900nm. Please note that ‘PL’ & ‘CH’ indicates plasmonic and chiral object respectively.

3. It is suggested to substitute all the line plots in Fig. 8 with higher resolution figures with readable values on the axes.

Ans: Dear reviewer, thank you for pointing out the clarity issue in Fig. 8. We have acted upon your recommendation and enhanced the resolution to as better as possible to showcase the finer details.

Fig. 6: (a) Near field OBF between plasmonic (gold) & chiral (red) objects immersed in half air-water and the setup is illuminated by x polarized counter propagating type waves (15-degree angle from the air-water interface). Distance between the particles is set to 200nm (b) F_y_ force on the plasmonic object from the dimer setup of (a). (c) F_y_ force on chiral object from the dimer setup of (a). (d) F_y_ force acting on a single half immersed plasmonic object for single light source (pushing force) (e) Fy force acting on a single half immersed chiral object for single light source (pushing force) (f) F_y_ force acting on a single half immersed plasmonic object with two light sources. This setup is illuminated by x polarized counter propagating type waves (15-degree angle from the air-water interface). (g) F_y_ force acting on a single half-immersed chiral object with two light sources. This setup is illuminated by x polarized counter propagating type waves (15-degree angle from the air-water interface). (h) Far field OBF between plasmonic (gold colored) & chiral (red colored) objects immersed in half air-water and the setup is illuminated by x polarized counter propagating type waves (15-degree angle from the air-water interface). Distance between the particles is set to 700nm (i) F_y_ force on plasmonic object from the setup of (h). (j) F_y_ force on chiral object from the setup of (h).

Reviewer #3

The manuscript prepared by SUDIPTA BISWAS et al. reported a repulsive force between plasmonic and chiral particles in both near and far field regions when counter-propagating plane waves are illuminated to the particles. But, in my opinion, the manuscript is not mature, so it is not easy to read and understand. The structure of the text is not well organized and scattered for me. The Introduction is too long. I do not feel the need to use the analog of two cars and two persons. Authors repeat the same things multiple times. More critically as a scientific report, some simulation details and results are not fully mentioned and/or explained in the text, for example, Figure 3b, 3c, and Figure 5. Especially Figure 3b, OBF is repulsive even though a single propagating wave is irradiated on the plasmonic-chiral heterodimer, which seems not consistent with author’s claim in this contribution. The discussion regarding to the wave and particle natures of light for the counterintuitive repulsive force (more specifically about so-called three catalysts) is not well examined in the manuscript. Thus, I do not recommend the manuscript to be published in the PLOS One. I strongly suggest the authors consider resubmitting after a basic rearrangement of the manuscript and get proofreading.

Ans: Dear Reviewer, Thank you so much for your valuable comments and constructive criticism. We have extensively revised and restructured the manuscript, eliminating redundant information in the introduction. We have colored red for our newly revised part in the manuscript. The car analogy has also been removed from the main manuscript. We identified an error in Figure numbering on line 211; it should be Figure 3, not Figure 4, and this issue has been rectified.

Regarding Figure 3(b), we observed a repulsive behavior when a single linearly polarized light propagating in the -y direction was applied to the chiral structure. However, with this setup, we were unable to reverse the longitudinal optical binding force (OBF). Additionally, when a single -y propagating light illuminated the dimer setup, we observe1d a pushing force for both Plasmonic and Chiral nanoparticles, as shown in the figure below. Nevertheless, in our primary setup, involving two counterintuitively polarized lights, we observed a reversal in the individual force of the chiral nanoparticle (cf. Supplement figure S2(a)), which is not true for Figure 3(b).

Similarly, in Figure 3(a), when light propagating in the +y direction illuminated the setup, we observed an overall attractive force. However, even in this case, the chiral nanoparticles exhibited a pushing force when the individual force is considered. In contrast, our primary setup demonstrated a reversal in the individual force for the chiral nanoparticles.

An essential objective of our paper was to control the overall longitudinal OBF in both near and far fields. Our final setup in Figure 4(a) indeed allowed us to achieve both repulsion and attraction, fulfilling this aim.

**A short discussion to clarify the associated wave-particle duality:**

**Wave Nature:**

From the revised manuscript:

“Certainly, the presence of the other particle in the dimer set is impacting the dynamics of another particle. The generic approach to explain such dynamics based on well-known dipolar force model [35] for the ‘simple’ dipoles:

In this model, the incident field induces a dipole in both the left-side and right-side particles, with the right-side dipole oscillating with a phase shift kd (where d represents the interparticle distance) relative to the left-side one. The right-sided dipole then radiates backward toward the left sided dipole. This backward-scattered light reaches the oscillating left sided dipole with the total phase shift of 2kd. The force on the left sided particle arises from the interaction of the left sided dipole with the interference effect of the backward-scattered field and incident field. On the other hand, the force on the right sided particle pushes the right sided dipole along the wave propagation direction. In this way, the interaction between both ‘simple’ dipoles is usually repulsive in the very closely placed near field region.”

- This clearly supports the wave nature of light.

**Particle Nature:**

From the revised manuscript:

“For our case, the plasmonic particle is experiencing pulling force (towards its nearest unperturbed light source) in the dimer set-up of Fig. 8(a) for the combined impacts of three catalysts together: (i) the presence of high non-paraxial angled beams (that creates multipolar response), (ii) transfer of Minkowski momentum (of emitted photon) to the plasmonic particle and (iii) repulsive force in the near field in presence of the chiral particle due to mutual scattering. If any of the aforementioned catalysts is removed, mutual repulsion at higher wavelength region or individual pulling type force vanishes. In summary, the dimer set, illustrated in Fig. 8(b) and (c), experiences a repulsive binding force even in higher wavelength regions, as depicted in Fig. 8(a), due to the pulling force exerted on each particle. Here, the pulling force refers to the force directing a particle towards the closest unperturbed light source.”

- The Minkowski momentum of photon suggests the linear increase of momentum of light at the interface of the half-immersed object and the background [A]. This increase of the linear momentum of light strongly suggests the existence of photon or the particle nature of light. An experimental observation in favor of such linear increase of momentum of photon for the Mie particle (but not Rayleigh or Dipolar particle) can be found in [B] and its initial analysis can be found in [A, C].

[A] Mahdy, M. R. C., Hamim Mahmud Rivy, Ziaur Rahman Jony, Nabila Binte Alam, Nabila Masud, Golam Dastegir Al Quaderi, Ibraheem Muhammad Moosa, Chowdhury Mofizur Rahman, and M. Sohel Rahman. "Dielectric or plasmonic Mie object at air–liquid interface: The transferred and the traveling momenta of photon." *Chinese Physics B* 29, no. 1 (2020): 014211.

[B] Kajorndejnukul, Veerachart, Weiqiang Ding, Sergey Sukhov, Cheng-Wei Qiu, and Aristide Dogariu. "Linear momentum increase and negative optical forces at dielectric interface." Nature Photonics 7, no. 10 (2013): 787-790.

[C] Qiu, Cheng-Wei, Weiqiang Ding, M. R. C. Mahdy, Dongliang Gao, Tianhang Zhang, Fook Chiong Cheong, Aristide Dogariu, Zheng Wang, and Chwee Teck Lim. "Photon momentum transfer in inhomogeneous dielectric mixtures and induced tractor beams." Light: Science & Applications 4, no. 4 (2015): e278-e278.
